# Soil iron and aluminium concentrations and feet hygiene as possible predictors of Podoconiosis occurrence in Kenya

**DOI:** 10.1371/journal.pntd.0005864

**Published:** 2017-08-23

**Authors:** Jacinta Muli, John Gachohi, Jim Kagai

**Affiliations:** 1 School of Public Health, Jomo Kenyatta University of Agriculture and Technology, Nairobi, Kenya; 2 Washington State University–Global Health Kenya, University of Nairobi Institute of Tropical and Infectious Diseases, Kenyatta National Hospital Campus, Nairobi, Kenya; 3 Centre for Biotechnology Research and Development, Kenya Medical Research Institute, Nairobi, Kenya; George Washington University School of Medicine and Health Sciences, UNITED STATES

## Abstract

**Background:**

Podoconiosis (mossy foot) is a neglected non-filarial elephantiasis considered to be caused by predisposition to cumulative contact of uncovered feet to irritative red clay soil of volcanic origins in the tropical regions. Data from structured observational studies on occurrence of Podoconiosis and related factors are not available in Kenya.

**Methodology/Principal findings:**

To establish the occurrence and aspects associated with Podoconiosis, a cross-sectional survey was implemented in an area located within 30 km from the foot of volcanic Mount Longonot in the Great Rift Valley in Kenya. Five villages and 385 households were selected using multistage and systematic random sampling procedures respectively during the survey. Podoconiosis was determined by triangulating (1) the clinical diagnosis, (2) molecular assaying of sputum samples to rule out *Wuchereria bancrofti* microfilaria and (3) determining the concentration of six elements and properties in the soil known to be associated with Podoconiosis. A structured questionnaire was used to identify possible risk factors. Univariable and multivariable Poisson regression analyses were carried out to determine factors associated with Podoconiosis. Thirteen participants were clinically positive for Podoconiosis giving an overall prevalence of 3.4%. The prevalence ranged between 0% and 18.8% across the five villages. Molecular assay for *W*. *bancrofti* test turned negative in the 13 samples. The following factors were positively associated with the Podoconiosis prevalence (*P*<0.1) in the univariable analyses: (i) age, (ii) gender, (iii) education level, (iv) frequency of washing legs, (v) frequency of wearing shoes, (vi) soil pH, and (vii) village. Unexpectedly, the concentration of soil minerals previously thought to be associated with Podoconiosis was found to be negatively associated with the Podoconiosis prevalence (*P*<0.1). In the multivariable analyses, only frequency of wearing shoes and village turned out significant (*P*≤0.05). By modeling the different soil mineral concentrations and pH while adjusting for the variable frequency of wearing shoes, only iron concentration was significant and in the negative dimension (*P*≤0.05). However, controlling for Iron, Aluminum concentrations turned significant.

**Conclusion/Significance:**

This study has pointed to a hitherto unreported occurrence of Podoconiosis cases and has contributed to the baseline knowledge on the occurrence of Podoconiosis in Kenya. Consistent with many studies, wearing shoes remain an important risk factor for the occurrence of the disease. However, our findings are inconsistent with some of the hitherto postulations that associate Podoconiosis prevalence with certain minerals in the soil in other regions in Africa. These findings provide new beginnings for the cross-disciplinary research of Podoconiosis in environmental health, socio-ecology and ecological niche and geo-spatial modeling and prediction.

## Author summary

Podoconiosis is a neglected disease in the tropical regions of the world considered to be caused by prolonged contact of uncovered feet to irritant particles found in red clay soil from volcanic origins. The disease presents like filarial elephantiasis. Data from observational studies from Kenya are not available. We conducted a cross-sectional household survey to establish the prevalence and aspects related with Podoconiosis at the foot of Mount Longonot in the Great Rift Valley in Kenya. Podoconiosis was determined by combining results of clinical diagnosis, ruling out filarial elephantiasis in clinically positive Podoconiosis patients using molecular techniques and determining the concentration of elements and properties in the soil known to be associated with Podoconiosis. A structured questionnaire was used to identify possible risk factors. Out of 385 study participants, thirteen were clinically positive for Podoconiosis giving an overall prevalence of 3.4%. Molecular tests for filarial elephantiasis turned negative in the 13 participants. Factors that were associated with Podoconiosis prevalence were age, gender, education level, and frequency of washing legs, frequency of wearing shoes, soil pH and village. The concentration of soil minerals previously thought to be associated with Podoconiosis was found to be negatively associated with the Podoconiosis prevalence. However, the final analyses found frequency of wearing shoes, iron and aluminium as possible predictors of Podoconiosis occurrence in the study area. This is the first structured observational study to report occurrence of Podoconiosis in Kenya. Although some of our findings are inconsistent with some previous reports about the association of Podoconiosis and certain minerals in the soil, this study offers new beginnings for the cross-disciplinary research of Podoconiosis in fields known to influence occurrence of the disease including environmental health, socio-ecology and medical geographical approaches and predictions.

## Introduction

Podoconiosis is a neglected geochemical, non-filarial, non-infectious lymphodema of the lower limb [1]. In Africa, countries in which non-filarial elephantiasis have been reported include: Tanzania [2], Uganda [3], Kenya [4], Cameroon [5], Sao Tome and Principe [6], Rwanda, Burundi, Sudan, Ethiopia [7], and Equatorial Guinea [8]. Podoconiosis is known to result from an interaction between genetics and an unusual provocative response to reactive mineral particles found in clay soil, red in color, derived from volcanic origin deposits [1]. Mineral particles from the soil are thought to penetrate the skin, then they are fought by macrophages in the lymphatic system which causes inflammation and fibrosis of vessel’s lumen leading to blockage of the lymphatic drainage [9]. This results in edematous feet and legs and subsequently progresses to elephantiasis [10] and nodular skin changes [11].

The prevalence of the disease is reported to vary considerably from country to country. For instance, reports show an average burden of 1% (range: 0% to 2.1%) in Burundi [12] and 0.6% (range: 0.1% to 1.7%) in Rwanda [12]. Other reports indicate that prevalence ranged from 0.4% to 3.7% in Ethiopia [13]. Recently, higher prevalences (range: 3.3 to 7.4%) have been reported in Ethiopia [14][15][16][17]. Non-filarial elephantiasis cases were also documented in Kenya at the foot of Mt. Kenya in the year 1948 [18]. However, there are no published findings from structured observational studies in Kenya.

Numerous structured studies investigating the role of individual-level risk factors have been carried out in Ethiopia. Gender, age, marital status, feet hygiene, level of job skills/employment, education level and house floor type have been associated with risk of Podoconiosis. Being older [15], female, single, rarely washing feet, and low skilled or jobless [19][20] showed significant association with increased occurrence of Podoconiosis. On the other hand, formal education [20] and living in a house whose floor is covered [20] were related with low risk of Podoconiosis. These findings point to the opportunities of modifying certain risk factors in the prevention of the disease.

Podoconiosis has been reported to be prevalent in highlands of tropical Africa, Central America and Northwest India all characterized by certain soil types [11][21]. Altitude and rainfall are among other factors which has been associated with occurrence of Podoconiosis [20]. These geographic characterizations are associated with consistent breakdown of molten rock and their mineral components into silicate clays. These geologic properties, through the development of peripheral water gradient potentially influence permeability of the stratum corneum in the skin and raise transdermal uptake of potential toxins and colloid-sized particles of elements common in irritant clays [10]. Hence, Podoconiosis is widespread in countries which are within the Rift Valley geological complex in Africa [11][12] and other areas with volcanic soils [22]. Soils within these areas are red clay loams, slippery and stick to the skin when wet [11]. Here lies the opportunity of utilizing the African Soil Atlas by specifically predicting Podoconiosis occurrence (http://eusoils.jrc.ec.europa.eu/content/soil-map-soil-atlas-africa) in Africa.

Moreover, Podoconiosis is occupational in nature with familial inclination in addition to a deficiency in feet hygiene. Podoconiosis occurs among farmers and other occupational groups whose feet remain uncovered and exposed to clay soil originating from alkaline volcanic rock [22]. Small particles such as silica, sodium, magnesium, aluminum, iron and potassium [12][23][24] of the incriminated soil type (almost nanoparticles) are thought to pass through the skin cracks and find their way into the lymphatic system. Besides, studies have shown high hereditability of susceptibility to Podoconiosis [11][25]. Indeed, the prevalence was reported to be higher in people who rarely wore shoes, indicating possible interrelationship between Podoconiosis, genetics, occupation, environmental factors and lifestyle [12].

Podoconiosis is associated with a host of disease burdens. The quality of life is substantially reduced [26][27]. Though non-fatal, those affected will show spoiled appearance of their legs [28]. Clinically, most patients acquire repeated infections of bacterial and fungal nature in the affected leg(s) necessitating extra medical attention [15][29]. Approximately all Podoconiosis patients suffer from acute lymphadenitis five or more times a year. It has been estimated that they lose an average of one month of economic activity every year due to morbidity [15][29][30]. In Southern Ethiopia, an assessment of the economic costs of Podoconiosis indicated that, in an area with 1.7 million residents, the cost of the disease was 16 million US Dollars annually, hence leading to Ethiopian loss of 200 million US Dollars per year. A research comparing affected and unaffected people within the same level of employment showed that those with disease are half as productive as those without disease. [31]. Stigma associated with Podoconiosis is manifested in people by dropping from schools, exclusion from social community activities, diminished marriage opportunities and reduction in economic development and psychological trauma [32].

Diagnosis of Podoconiosis is not straightforward. To rule in Podoconiosis, geographical location, history, clinical findings and confirmed absence of microfilaria or its antigen on immunological card test are used. Geographically, it has been found that Podoconiosis is prevalent in populations who live at high altitudes (>1000 metres above sea level). Clinically, Podoconiosis is an ascending and commonly bilateral non-filarial elephantiasis though asymmetric [33] and rarely involves the groin. On the other hand, lymphatic filariasis is found at altitudes lower than 1000 metres above sea level. In addition, clinical changes are first noticed at the groin in lymphatic filariasis. We used these features to triangulate the diagnosis of the disease.

This paper reports a cross-sectional household survey implemented to establish the burden and factors related with Podoconiosis occurrence. This information will be useful to the health administrators and humanitarian agencies responsible for developing and implementing targeted, appropriate and effective public health intervention strategies. This is the first field-based observational survey that has acknowledged the occurrence of Podoconiosis in Kenya.

## Materials and methods

### Study site

The study was carried out in Mt. Longonot region in Nakuru County with a population of 1,603,325 (year 2009 census) [34] and an area of 2,325.8 km^2^. Mount Longonot is located within Nakuru County, Kenya which is an agriculturally rich county. It is also a strato-volcano situated southeast of Lake Naivasha in the Great Rift Valley of Kenya in Africa. The county generally has an elevation of 2776m [35]. Nakuru County has temperatures ranging from a minimum of 12°C to a maximum of 26°C. Rainfall ranges from 1800 to 2000mm per year [36].

### Study design and population

A cross-sectional quantitative community-based household survey was implemented in this study. The study population consisted of women, men and children aged 5 years and above from the area of study. This age was chosen because Podoconiosis incidence rises with age [37]. We included people who were residents of the study area, had lived in the study area for five or more years and also consented to take part in the study. The exclusion criteria were: those aged less than five years, not have lived in the area for five years and above and those who did not consent to the study.

### Sample size determination and sampling procedure

The sample size was calculated using the Cochran formula [38]. Due to unavailability of the prevalence of Podoconiosis in Nakuru County, 50% prevalence and a tolerable error (level of precision) of 5% was used to determine the sample size. A sample size of 385 participants was computed using the formula below;

$$n=\frac{1.96^{2}p\;(1\text{-}p)}{L^{2}}$$

Whereby, 1.96 was the *z* value for the desired confidence level (95%), *p* was an estimate of the probable prevalence of Podoconiosis and *L* was the level of precision. Multi-stage random sampling was used to select villages to be included in the study. Villages within 30 km from the foot of Mt. Longonot were identified. Nine villages were identified and five of them selected randomly. Those selected included Githarani, Scheme, Ereri, Lower Kiambogo and Upper Kiambogo.

The number of study participants in each village proportionately depended on the village population and the calculated sample size and was computed as shown below:

$$n_{i}=\frac{N_{i}}{N}*n$$

Where *n*_*i*_ was the sample size for a village *i*, *N*_*i*_ was the population size in village *i*, *N* was total population in the study site, and *n* was the overall calculated sample size (385). Table 1 shows the computed statistics by village with *N* being 6068. One participant was randomly selected in each household; therefore, the number of participants was equal to the number of households to be included in the study. Systematic selection of the households was done depending on the total number of households to the sample households required from each village by dividing the number of households in each village by sample size in that village (Table 1). For instance, from any starting point, Githarani, households were selected at the interval of every three houses. In case the third house did not have residents, we selected the next one that had residents. The study participant was subsequently selected randomly from the present household members.

**Table 1:** Sampling distribution by village.

Village	*N*_*i*_	*n*_*i*_	Number of households in village	Sampling interval
Githarani	500	32	99	99/32 = 3.09
Ereri	1005	64	99	99/64 = 1.54
Lower Kiambogo	493	31	86	86/31 = 2.77
Scheme	70	4	20	20/4 = 5
Upper Kiambogo	4000	254	400	400/254 = 1.6
**Total**	**6068**	**385**	**704**	

### Soil sampling and analyses

With the help of research assistants, one soil sample was collected from each of the five villages giving a total of five soil samples. Each unique sample consisted of cores taken from study households and pooled together within each village. Soil was dug using a 12cm shovel to a depth of 25cm since the elements occur on the surface layer of between 0-25cm. The amount dug was placed in a bucket and thoroughly mixed. Moist soil samples were air dried at the site away from dust contamination. The soil sample bags which were well labeled with the sample code were filled half full (500g) from this mixed representative sample and tightly packed [39]. The samples were kept in a secure room at room temperature and transported as a batch to SGS Laboratories in Mombasa, Kenya.

Soil analyses was done at SGS Laboratories in Kenya to provide data on the level of concentration of the following elements—aluminum, magnesium, silica, sodium, potassium and iron, and pH- known to be associated with Podoconiosis [24]. Atomic Emission spectrometry (Spectro Flame Modula ICP) instrument using Mehlich 3 –Diluted ammonium fluoride and ammonium nitrate [40] was used for analyses. Briefly, the soil samples were dried for 72 hours at 150°F and then crushed in a Dynacrush soil crusher to a size it can pass a 20-mesh screen. Two grams of the soil sample was scooped into 70ml extraction cup made of plastic in a Styrofoam rack. A pipette machine was used to add 14ml of Melich 3 extractant to the extraction cup. The extraction cups, held in a plastic rack were positioned in the Eberbach shaker. Above the tops of the extraction cups, a sheet of plastic cover was positioned and shaker lid closed and protected to hold the racks firm. For 5 minutes, a shaker power cord was connected to a power output plug on a GRA lab timer. Later, in the filter tube in the wooden filter rack, an 11 cm #1 of filter paper was put in place. The cup holder (rack) was removed when the shaker stopped and poured into filter papers. The sample was then transferred to autosampler tubes for analysis using Inductively Coupled Plasma (ICP) spectrometry.

### Household data collection tools and processes

Data was collected for one month by the lead author and three field officers. One day prior to the survey, a brief meeting was held with the field officers to give details regarding the study. The clinical officer used a clinical investigation form and physical examination of legs to diagnose Podoconiosis. Clinical characteristics and symptoms included bilateral but asymmetrical swelling of legs below the knee, itching and burning episodes of lower legs, lack of tropic ulcers and availability of sensation. Sputum samples from clinically positive participants were collected for molecular assay to detect *Wuchereria bancrofti* which causes filariasis. This was carried out to rule out filariasis which presents like Podoconiosis. The molecular assaying was executed at Kenya Medical Research Institute at the Centre for Biotechnology, Research and Development. The molecular assaying is reported to have a sensitivity of 97.5% and specificity of 92.4% [41]

Social-demographic information was collected using structured questionnaire and included age, sex, marital status, education level, type of house floor, frequency of wearing shoes, occupation and level of hygiene.

### Molecular assay of sputum samples

Sputum samples were collected in sterile containers for PCR to rule out *Wuchereria bancrofti* which causes filariasis. The samples were kept in well-sealed cooler boxes containing ice packs and transported to the nearby hospital for storage for two weeks before being transported to KEMRI.

Approximately 200μl of sputum (mucoid) was used to extract *Wuchereria bancrofti* DNA. All procedures were done on ice. DNA was extracted from sputum samples by alkaline precipitation method as described by Zhong et al. [42]. Approximately 200μl of serum sample was added into sterile eppendorf tubes. 198μl of 1% Triton and NaOH was added and the mixture vortexed. The mixture was heated at 65°C for 30 minutes in a thermo mixer. The PH was adjusted to 8 with HCl 1:4 or 1M NaOH. The mixture was spinned quickly at 4°C, 14000 RPM for 5 minutes and the supernatant transferred into clean Eppendorf tubes. It was heated at 100°C for 5 minutes in a thermo mixer and subsequently cooled quickly on ice. Every tube was added four hundred (400) μl of absolute ethanol and kept at 70°C in the freezer for overnight. For 20 minutes, they were spinned at 4°C, 14000 RPM and the supernatant was discarded by pipetting 400μl. It was then washed thrice with 70% ethanol. They were then spinned in a micro-concentrator for 1 hour. The mixture was suspended in 50μl of TE buffer and vortexed and stored at -20°C until PCR was carried out.

Ten (10) μl of the extracted DNA was used for DNA amplification to enable detection of *Wuchereria bancrofti* DNA for diagnosis of filariasis. This was done using a thermo cycler machine. A master mix preparation was first prepared as shown in Table 2. The master mix (Table 2) was first vortexed. 50μl of the master was put to a well labelled PCR tubes and 10μl of the sample added. 0.5μl of Taq polymerase was also added to the sample and the tubes positioned into a thermo cycler with the programme set and allowed to run for 35 cycles. The amplified DNA (template) samples, molecular marker, positive and negative controls were loaded in 2% agarose gels in the gel electrophoresis tank and allowed to run for one hour. It was then visualized by UV illuminator and the samples viewed against 200 base pairs in the molecular marker.

**Table 2:** Master mix preparation.

Reagent	Final conc. in PCR Per reaction-1X
10X PCR Buffer	5μl
5pmol direct primer	0.5 μl
5pmol reverse primer	0.5μl
100mM dNTPs mix	0.5μl
PCR water	43.5 μl
Total reaction Volume per tube	50 μl
Taq DNA polymerase	0.5μl- (to each reaction tube)

### Data analysis

Data was entered into computer Statistical Package for Social Sciences (SPSS) software v.20.0 for analysis. Descriptive analyses were initially carried out. The study outcome was computed as the proportion of the sample surveyed that had clinical Podoconiosis. As this proportion was very small (see results below), the outcome was assumed to represent a count of the number of cases in the group. To relate the count of the cases to predictors (socio-demographic and soil mineral concentrations), a Poison regression model was assumed in the form: $\ln\frac{E(Y)}{\eta }=\beta_{0}+\beta_{1}X$ where the term on the left of the equation was the log of the expected value of counts of disease which was modelled as a linear combination of the predictors (on the right of the equal sign). The model related the log of the expected value of counts of disease and a linear combination of one predictor in univariable analysis at a significance level of *P*≤0.1. The model was subsequently extended to control for other predictors by including all significant variables at the univariable step in multivariable analyses at a significance level of *P*<0.05 as follows:

$$\frac{E(Y)}{\eta }=\beta_{0}+\beta_{1}X_{1}+\beta_{2}X_{2}+\ldots \ldots \;\beta_{k}X_{k}$$

Where *k* was the number of predictors.

Multivariable modeling was carried out by backward elimination strategy and, in addition, involved checking of confounding and relevant interaction terms. During modelling, the statistical significance of the contribution of individual predictors (in univariable analyses) or groups of predictors (in multivariable analyses) to the model was tested using the likelihood ratio test.

The models were assessed for overall fit using *χ*^2^ goodness-of-fit tests computed as the sum of the squared deviance or Pearson residuals. The values of the two test statistics were compared (as they can be quite different) to assess lack of fit. As with all overall goodness-of-fit statistics, a *P*>0.05 (non-significant) indicates that the model fits the data well.

### Ethics statement

This study was registered under Kenya Medical Research Institute (KEMRI), and was approved by Scientific Ethical Review Committee, KEMRI number KEMRI /RES/7/3/1. Informed consent was obtained from each study participant after reading and or providing a detailed oral explanation to all potential participants with the help of an assistant research officer. The participants were given a chance to decide whether to voluntarily participate in the study. Those willing to join the study were requested to sign on the consent form. Those with reduced capacity to sign were allowed to put a thump print on the consent form to prove consent. In individuals aged below 18 years, informed consent was obtained and interview conducted to the parents or legal guardian and the process continued as above depending on whether they were able to put a signature or thumb print on the consent document. In addition, there was a verbal assent between the researcher and children aged 13–17 years. Participants between 13–17 years of age, who accepted to participate in the study signed or put a thump in the assent form. The research assistant and the principal investigator signed the consent form as witnesses. The process of oral informed consent as well as the consent procedure was approved by the Scientific Ethical Review Committee, KEMRI.

## Results

### Social demographic characteristics of participants

A total of 385 participants aged 5 years and above were included in the study. The participants had a mean (standard deviation) age of 44.8 years (19.8). The sample comprised of 108 (28.1%) males and 277 (72.0%) females. Most of these participants were aged between 29 and 38 (20.5%) years old. Most of the study participants had formal education (72.0%). Of the study participants, 65.2% lived with a spouse while 34.8% lived without a spouse. Majority of the study participants were farmers; *n* = 291 (75.6%). More than 50% of the participants had their house floor made of earth. In addition, most of the participants washed their legs daily (99.5%) and wore shoes daily (76.9%) as shown in Table 3.

**Table 3:** Social demographic characteristics of participants (n = 385).

Variable	Category	Frequency	Percentage (%)
**Age group**	**5–17**	**20**	**5.2**
	**18–28**	**72**	**18.7**
	**29–38**	**79**	**20.5**
	**39–48**	**64**	**16.6**
	**49–58**	**51**	**13.3**
	**59–69**	**33**	**8.6**
	**70 and above**	**66**	**17.1**
**Gender**	**Male**	**108**	**28.0**
	**Female**	**277**	**72.0**
**Educational level**	**No formal education**	**67**	**17.4**
	**Formal education**	**318**	**82.6**
**Marital status**	**Living with spouse**	**251**	**65.2**
	**Not living with spouse**	**134**	**34.8**
**Type of occupation**	**Farming**	**291**	**75.6**
	**Others**	**94**	**24.4**
**Type of floor**	**Non-earth**	**160**	**41.6**
	**Earth**	**225**	**58.4**
**Frequency of washing legs**	**Rarely**	**2**	**0.5**
	**Daily**	**383**	**99.5**
**Frequency of wearing shoes**	**Rarely**	**89**	**23.1**
	**Daily**	**298**	**76.9**

### Soil element concentration and PH

The concentration of silicon, aluminium, sodium, iron, potassium and magnesium was determined in five soil samples—each sample from each study village. The pH was also determined and the results shown in Table 4. The mean values of silicon, aluminium, sodium, iron, potassium and magnesium were 186.99mg/kg, 10303.82mg/kg, 264.45mg/kg, 15011.95mg/kg, 2121.99 mg/kg and 787.81mg/kg respectively. The mean PH was 7.04 as illustrated in Table 5.

**Table 4:** Distribution and concentration of soil elements in mg/kg and soil PH in soil sampled in Mt Longonot area by village.

Village	Silicon	Aluminium	Sodium	Iron	Potassium	Magnesium	PH
**Githarani**	158	4079.45	268.34	8610.61	953.12	372.7	7.4
**Scheme**	160.02	5734.87	241.94	10586.2	875.47	342.9	6.4
**Ereri**	100.78	3995.13	216.16	12677.2	917.1	437.66	6.8
**Lower Kiambogo**	159.68	11611	266.96	14414.6	3496.87	1331.43	7.4
**Upper Kiambogo**	216.12	12590	276.17	16549.3	2424.68	869.46	7.0

**PH**-Potential of Hydrogen **mg/kg**-Milligrams per Kilogram

**Table 5:** Overall mean, standard deviation, minimum and maximum values of soil element concentration in mg/kg and soil PH in soil sampled in Mt Longonot area.

Element	Mean	Standard deviation	Minimum	Maximum
**Silicon**	186.99	44.02	199.78	216.12
**Alluminium**	10303.82	3697.99	3995.13	12,590
**Sodium**	264.45	22.06	216.16	276.17
**Iron**	15011.95	2473.75	8610.61	16549.3
**Potassium**	2121.99	764.99	875.47	3496.87
**Magnesium**	787.81	255.094	342.9	1331.43
**PH**	7.03	0.19	6.4	7.4

**PH**-Potential of Hydrogen; **mg/kg**-Milligrams per Kilogram

### Prevalence of Podoconiosis and univariable analysis

**Fig 1:**
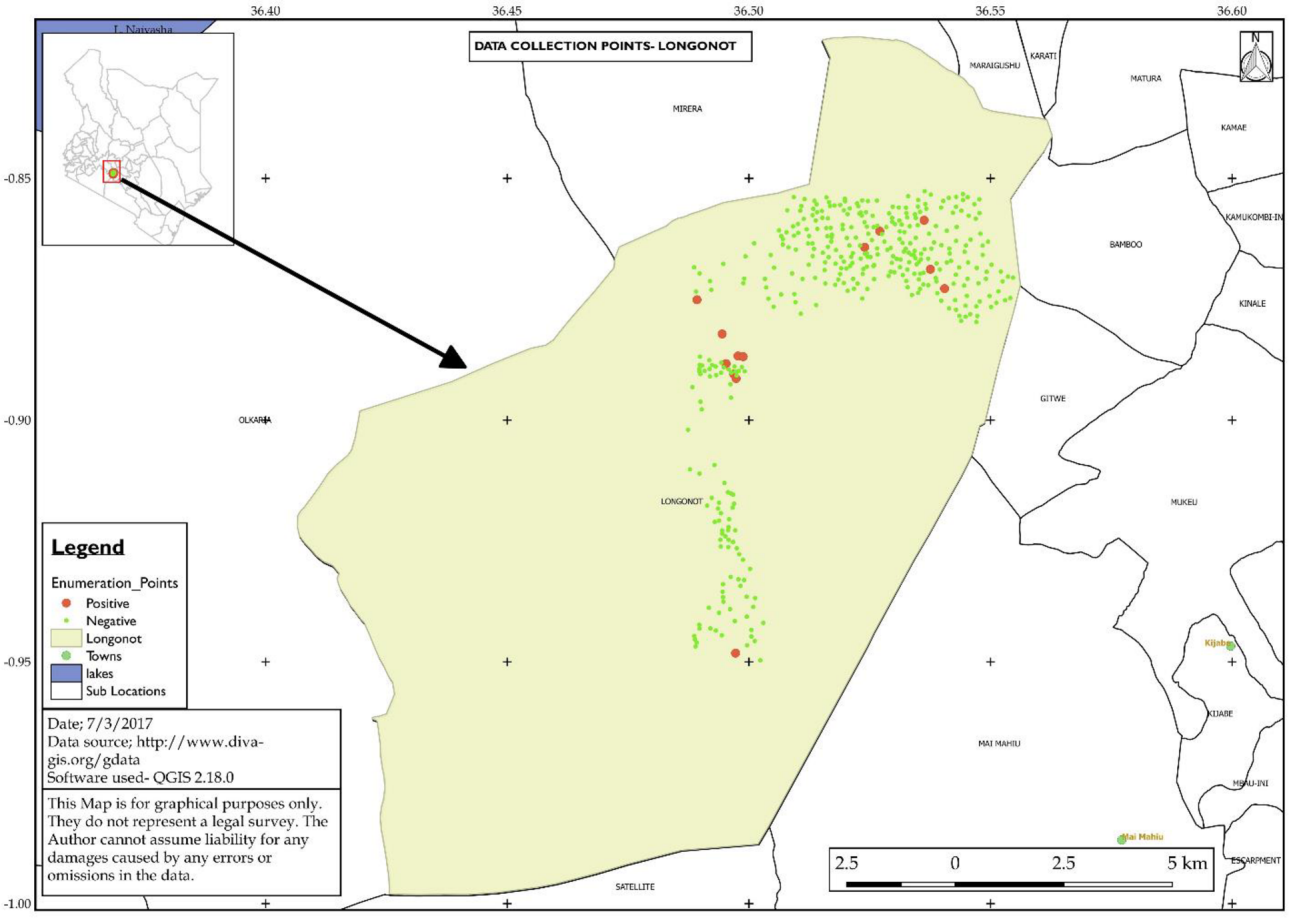
Study site showing the selected study households. Big red dots indicate clinically positive households while the small green dots indicate clinically negative households. Top left hand is a map of Kenya showing the location of Mt Longonot area.

Of the total sample size, 13 (3.4%, [95% CI, 1.8%, 5.7%]) were found to be clinically positive for Podoconiosis. Fig 1 shows the distribution of households with clinically positive and negative study participants. Table 6 shows univariable analyses of social-demographic and socio-economic factors, village, soil pH and soil element concentration associated with Podoconiosis. The prevalence ranged between 0% and 18.8% across the five villages with Githarani, Scheme, Ereri. Lower Kiambogo and Upper Kiambogo reporting 18.8%, 0.0%, 1.6%, 3.2% and 2.0% respectively (Table 6). Univariable analyses screened 16 variables but returned 12 significant variables (*P*≤0.1) associated with the count of Podoconiosis in the study area. These variables included age, gender, education level, and frequency of wearing shoes, village of residence and frequency of washing legs.

**Table 6:** Counts and prevalence of Podoconiosis and univariable analyses relating the counts of Podoconiosis and risk factors collected in Mt Longonot area, Kenya.

Factor	Level	Positive	Prevalence (%)	Coefficients [95% CI]	Likelihood P-value
**Age**				0.04[0.020, 0.660]	0.001
**Gender**	Female	12	4.3	Ref	
	Male	1	0.9	-1.5[-3.54, 0.55]	0.08
**Marital status**	With spouse	8	3.1	Ref	
	Without spouse	5	3	0.16[-0.91, 1.33]	0.72
**Education level**	Formal	8	2.5	Ref	
	Non-formal	5	7.5	1.09[-0.11, 2.19]	0.07
**Occupation**	Other	2	2.1	Ref	
	Farming	11	3.8	0.40[-0.91, 2.27]	0.58
**Floor Type**	Non-earth	3	1.9	Ref	
	Earth	10	4.4	0.77[-0.39, 2.29]	0.20
**Frequency of wearing shoes**	Daily	2	0.7	Ref	
	Rarely	11	12.4	2.96[1.65,4.83]	0.00
**Frequency of washing legs**	Non-daily	1	50	Ref	
	Daily	12	3.1	-2.92[-4.8, 0.73]	0.05
**Village**	Githarani	6	18.8	Ref	0.0002
	Scheme	0	0	-12.6[-1925.7, 1900.5]	
	Ereri	1	1.6	-2.8[-4.91, -0.67]	
	Lower Kiambogo	1	3.2	-1.75[-3.87, 0.36]	
	Upper Kiambongo	5	2	-3.15[-4.34, -1.97]	
**Silicon**				-0.11[-0.02, 0.001]	0.06
**Aluminium**				-0.0002[-0.0003, 0.0001]	0.001
**Sodium**				-0.004[-0.02, 0.04]	0.78
**Iron**				-0.0004[-0.01, -0.0002]	0.00
**Potassium**				-0.001[-0.001, -0.0003]	0.005
**Magnesium**				-0.004[-0.006, -0.001]	0.003
**Soil PH**				5.95[3.31, 8.59]	0.00

Ref: variable category in the reference; CI: Confidence Interval

If a study participant were to increase the age by one year, the difference in the log of expected counts of Podoconiosis was expected to increase by 0.04 (Table 6), i.e. increased age was associated with Podoconiosis occurrence. The difference in the log of expected counts of Podoconiosis was expected to be -1.5 units lower for males compared to females (Table 6), i.e. Podoconiosis was more likely to be found in females relative to males. Additional risk factors (interpreted in the same way) included non-formal education, walking and working bare feet, failure to wash legs daily, and village (Table 6). In addition, the concentrations of silicon, aluminium, iron, magnesium and potassium were separately found to be significantly associated with the log of expected counts of Podoconiosis cases. However, this relationship was protective to the occurrence of Podoconiosis cases. Lastly, if the soil pH were to increase by one unit, the log of expected counts of Podoconiosis was expected to increase by 6 (Table 6), i.e. alkaline soil was a risk factor of Podoconiosis occurrence.

### Multivariable analysis

In the multivariable analyses (*P*≤0.05), only two variables of the 12 screened in the univariable model remained significant (Table 7). This included frequency of wearing shoes and village. The logs of expected counts of Podoconiosis was expected to be 2.7 units higher for study participants who rarely wore shoes compared to those who wore shoes daily while holding the village variable constant in the model (Table 7). In addition, the difference in the logs of expected counts of Podoconiosis was expected to be lower for study participants from all villages compared to study participants from Githarani village holding the wearing shoes variable constant in the model. The risk of Podoconiosis declined in this order in the villages: Githarani, Lower Kiambogo, Ereri, Upper Kiambogo and Scheme (Table 7).

**Table 7:** Significant variables in the multivariable (*P*≤0.05) model assessing relationship between log of expected counts of Podoconiosis and risk factors.

Variable	Level	Coefficient [95% Confidence interval]	P>|z|	Likelihood P-value
Frequency of wearing shoes	Daily	Reference	0.001	0.000
	Rarely	2.7 [1.1, 4.2]		
Village	Githarani	Reference		0.0020
	Scheme	-11.6 [-2885.7, 2862]	0.994	
	Ereri	-2.4 [-4.5, -0.29]	0.026	
	Lower Kiambogo	-1.5 [-3.6, 0.57]	0.153	
	Upper Kiambogo	-2.7 [-3.9, -1.5]	0.000	

### Multivariable analysis without the variable village

As soil samples had been collected from the villages, they could not be modeled together with the village variable. In the second set of modeling, the variable village was dropped and the different soil mineral concentrations (silicon, aluminium, potassium, magnesium and iron) and pH modelled while adjusting for the variable frequency of wearing shoes. This stage of modeling was implemented to tease out the effect of the village. The element concentrations represented the study villages since each soil sample represented one village.

Adjusting for frequency of wearing shoes, only the iron concentration was significant (*P*≥0.05) (Table 8). If the concentration of iron were to increase by 1 mg, the difference in the logs of expected counts of Podoconiosis would be expected to decrease by 0.0003 units, while holding the variable frequency of wearing shoes in the model constant.

**Table 8:** Parsimonious multivariable (*P*≤0.05) model for the log of expected counts of Podoconiosis.

Variable	Level	Coefficient [95% confidence interval]	P>|z|	Likelihood P-value
Frequency of wearing shoes	Daily	Reference	0.000	0.000
	Rarely	2.7 [1.2, 4.2]		
Iron		-0.0003 [-0.0005, -0.0001]	0.000	0.002

### Assessment of confounding and interaction of soil minerals

Extensive analyses were carried out first to assess confounding of all soil minerals in presence of iron. Interestingly, in the presence of iron, the effect aluminium concentration on Podoconiosis changed from negative dimension (in the univariable analyses) to positive. This suggested that iron and aluminium could be related and acting as confounders for each other. To investigate this finding further, an interaction term was generated by adding the cross-product term (iron concentration*aluminum concentration) and testing if the coefficient term was statistically significant. The interaction term was significant independently but not in presence of iron or frequency of wearing shoe in the multivariable analyses.

### Evaluating the parsimonious poisson regression model

The parsimonious model of the data is illustrated in the model in Table 8. To assess the overall fit of the model, the chi-square goodness-of-fit tests were computed as the sum of the squared deviance and Pearson residuals. The resulting test statistic has an approximately χ^2^ distribution in presence of multiple observations within each covariate pattern defined by the predictors in the model (if it is significant, it indicates lack of fit). For this data, Deviance statistic had a *P* = 1.0000 whereas the Pearson statistic had *P* = 0.17 indicating that the model fit the data well.

## Discussion

This study provided a preliminary but detailed quantitative assessment of prevalence and factors associated with Podoconiosis occurrence in Mount Longonot region in Nakuru County in the Great Rift Valley in Kenya. Data was collected and triangulated by clinical investigation, responses from a structured questionnaire, molecular assaying to rule out infectious elephantiasis and soil mineral concentration analyses. The findings strongly pointed to possible occurrence of Podoconiosis in the region with a prevalence of 3.4%. According to our knowledge, this is the first field observational research of Podoconiosis prevalence in Kenya. This prevalence was similar to recent prevalence reports in different Podoconiosis endemic areas in Ethiopia ranging from 3.3% in Debre Eliyas and 3.4% in Dembecha *woredas* [17] with overall prevalence being 3.3%. An extensive evaluation documented a national prevalence of 3.4% in Ethiopia [20] similar to our study using the elimination method for diagnosis, with older studies reporting a prevalence of 2.7% [13] and 2.8% [1] in Ethiopia. Some studies in Ethiopia and elsewhere show a high prevalence between 2.8% and 7.4% [14][15][43]. A study carried out in Uganda in high-risk communities reported a prevalence of 4.5% [3], whereas another in Cameroon reported a prevalence of 8.1% [7]. Variation between our prevalence value and values from other reports are most likely due to differences in sampling methods, sample sizes, location, time and the level of risk.

Consistent with published findings, frequency of wearing shoes and soil mineral concentration (particularly iron) were linked with burden of Podoconiosis in this study. These variables show effect after a long period of exposure to reactive alkaline volcanic soils [10][11]. Minute mineral particles enter into the skin due to long-term exposure of uncovered feet to the reactive soil. This triggers a provocative reaction in the lymphatic system which causes thickening and subsequent obstruction of lymphatic system [9]. Emerging evidence also suggests that genetic susceptibility may play a role [44]. Indeed, a study has estimated that an offspring from an affected parent is five times more likely to develop Podoconiosis compared to an individuals selected randomly from the general population [10]. This estimate was not only due to shared environment alone, but surveys indicate that 63% of Podoconiosis prevalence is attributed to inheritance of susceptibility [10]. An interaction between genetic susceptibility and irritation from mineral particles has not been studied and this is a front for future research.

However, under univariable analyses, increasing age, female gender, low education level, low frequency of wearing shoes, low frequency of washing legs, high soil pH and some soil elements showed a statistically significant relationship with the prevalence of Podoconiosis.

Age, gender and education level are individual-level variables widely reported to be associated with prevalence of Podoconiosis. Increasing age is expectedly associated with occurrence of Podoconiosis most likely because age is a proxy for exposure time (cumulative exposure). Previous work reported that the disease mostly develops in the agriculturally productive ages of 16 to 54 years which could explain the cumulated exposure [20][32]. Consistent with our study findings, being a female would increase the chances of an individual having Podoconiosis [20]. Gender differences may exist in terms of preventive behaviours such as shoe ownership and wearing practices, risk behaviours such as kitchen and farm gardening as well as access to personal resources such as socks and shoes [20]. New areas for research include possible differences in genetic susceptibility [25] and biological susceptibility [20] which may be hormonal-based and, in addition, how gender roles may modulate the risk of the disease [20].

In this study, formal education was associated with decreased risk of Podoconiosis. This shows similarity with research done in Ethiopia [20] which reported that secondary and higher education was associated with decreased risk of Podoconiosis. Moreover, another study in Ethiopia [45] showed that the illiterate participants were 11 times more likely to develop Podoconiosis relative to those who had secondary education and above. This implies that Podoconiosis occurrence can predict low education level. On the other hand, low education level can predict Podoconiosis occurrence. These findings may mutually reinforce in a positive feedback loop with low education level and Podoconiosis ultimately converging leading to stigma [15]. On the other hand, formal education may prevent the occurrence of the disease by default due to better lifestyle arising from formal employment or by design where educated people are better informed of soil-borne infectious and non-infectious sources of inflammation and mostly live on non-earthen floored houses. Although age and gender are non-modifiable factors, health promotion and education are essential in endemic areas to empower residents in the control over, and to improve their feet health.

Foot hygiene practices including low frequency of wearing shoes and low frequency of washing legs were associated with increased prevalence of Podoconiosis. This is consistent with known strategies of Podoconiosis prevention. At early stages, one is capable of managing Podoconiosis and stop further disease development by regular cleaning of feet and consistent wearing of shoes [11]. However, Deribe et al [20] reported that there was no association between wearing shoes and Podoconiosis in Ethiopia. The difference between our finding and Deribe et al [20] could be partly due to individuals with disease starting to protect their legs after Podoconiosis has developed because of lack of awareness and increased stigma. In Kenya, the opposite is true as the awareness is very little or non-existent. Deliberate water, hygiene and sanitation (WASH) education and promotion should be extended to include feet in addition to hand hygiene in endemic areas in Kenya. Specific interventions are currently being tried in the field in Ethiopia including ‘foot hygiene’ [46], hence similar measures need to be introduced in Kenya.

In this study, increasing soil pH was associated with increasing prevalence of Podoconiosis and some soil elements showed a statistically significant relationship with the prevalence of Podoconiosis. Our findings are consistent with observations that the disease occurs as a result of exposure to alkaline clay soils [12][47]. In turn, the determinants of soil formation and characteristics include environmental variables such as climate and geology including weathering of rocks. Ecologically, local properties of soil are very crucial in Podoconiosis development [48]. A recent survey in Ethiopia was carried out using historical data to explore the distribution and environmental variables of Podoconiosis [48]. In the latter study, high prevalence of Podoconiosis was noted in areas of altitudes more than 1500m above seas level, with rainfall more than 1500mm per year, and temperatures ranging between 19–21°C annually. The significant village effect found in our study most likely reflected micro-differences in environmental factors that have hitherto been reported. For instance, in 1984, Price [25] noted that Podoconiosis prevalence reduced to almost zero at a distance of 25km from the point of high red clay soil. Recent incremental knowledge showed that higher levels of minerals within soil such as mica, quartz and smectite affected the occurrence of Podoconiosis [49]. Such minerals aid in water uptake, accelerate Podoconiosis occurrence by inducing pathology in individuals’ body leading to acute adenolymphangitis, a cause of severe morbidity among those affected. An important finding in this research is that the hitherto assumption which relates the availability of compounds and elements found in soil and Podoconiosis incidence [22] was not consistent. Presence of such types of data allows for environmental risk mapping in a spatial analytical framework to produce maps of reported cases and environment-based maps of the areas at risk of Podoconiosis. This approach can be augmented with Ecologic niche modeling (ENM) whose principle is to evaluate the potential spatial distribution of species. In the same way, the ENM approach would be used to define and delineate the niche of Podoconiosis as well as foresee its probable geographic plus ecologic distribution by the analysis to determine the association between environmental variables [50] for targeted intervention. A study in Ethiopia by Deribe et al [51] has initiated this promising area for research of Podoconiosis.

Diagnosis of Podoconiosis was based on differential diagnosis as done in other studies [17][20][47]. These comprise of leprosy, filarial elephantiasis and mycetoma pedis which are associated with tropical lymphodema [47]. Clinical diagnosis has been reported to be precise in endemic settings [34]. However, as opposed to filariasis where the primary swelling can occur anywhere in the inferior extremities, Podoconiosis exclusively commence in the feet. Secondly, Podoconiosis mostly occurs in both legs, but the swelling can differ in size [49] while mycetoma and filariasis is mostly unilateral. Moreover, groin involvement which is mostly an indication of filarial elephantiasis is very rare in Podoconiosis. This study uniquely triangulated Podoconiosis clinical picture, responses from structured questionnaire, molecular assay to rule out infectious elephantiasis and soil mineral concentration analyses. Local eco-epidemiology can also be a clue to diagnosis as Podoconiosis is typically found in higher altitude areas with volcanic soil simultaneously with higher rainfall [51] whereas filariasis is uncommon at higher altitudes and other environments in which the mosquito vector of filariasis is less prevalent.

This study is not without limitations. The study utilized a cross-sectional study design. These findings therefore need to be interpreted with caution and further and more intensive cross-disciplinary studies are needed to authenticate them. This is because cross-sectional studies are subject to problems of undocumented confounders operating at different scales. Furthermore, the time-relationship between the factors and the disease is not known with such a design. However, in this case, a cross-sectional study design was appropriate as the disease is mostly non-fatal and the associated variables do not have a clear time-onset. Secondly, Podoconiosis is thought to cluster within families/households, partially associated with common environmental exposure or shared genetic susceptibility. This approach could have underestimated the prevalence in the region. However, ours was a cross sectional study in the region with a descriptive purpose with respect to the outcome and a set of risk factors. Additionally, we did not count any additional Podoconiosis suspected cases in the households that were recruited for the study. Future research in the region should investigate the existence of disease clustering at the household and the spatial level to increase the understanding of the disease mechanisms. Distinct study designs particularly case-control studies are also warranted as they are appropriate for studying rare conditions or diseases such as Podoconiosis.

## Conclusion

This study reports the occurrence of Podoconiosis in the Mt Longonot area in the Rift Valley in Kenya. Soil Iron and Aluminium concentrations and feet hygiene were identified as possible predictors of Podoconiosis occurrence in Kenya. Because of the possible spatial restriction of the exposure, external validity to other areas may not be feasible and, therefore, we restrict our conclusions to the target (source) population. The findings in this study gives the baseline knowledge regarding the occurrence of non-filarial elephantiasis in Kenya and providing a fresh beginning in cross-disciplinary research of Podoconiosis using socio-ecology, environmental health and ecological niche and geo-spatial modeling and prediction.

## Supporting information

S1 Checklist STROBE checklist. (DOC)
