# Supplementary material for: Soil iron and aluminium concentrations and feet hygiene as possible predictors of Podoconiosis occurrence in Kenya
Source: PLoS Negl Trop Dis. 2017 Aug 23;11(8):e0005864. doi: 10.1371/journal.pntd.0005864 (PMC5584976; doi:10.1371/journal.pntd.0005864)
Supplement: S1 Checklist — (DOC) [file pntd.0005864.s001.doc]

STROBE Statement—Checklist of items that should be included in reports of ***cross-sectional studies***

|  | Item No | Recommendation |
| --- | --- | --- |
| **Title and abstract** | 1 | (*a*) Indicate the study’s design with a commonly used term in the title or the abstract **[Within the title and method section of the abstract]** |
|  |  | (*b*) Provide in the abstract an informative and balanced summary of what was done and what was found **[see methodology/findings section of abstract]** |
| Introduction | | |
| Background/rationale | 2 | Explain the scientific background and rationale for the investigation being reported **[See introduction section]** |
| Objectives | 3 | State specific objectives, including any prespecified hypotheses **[refer to introduction section]** |
| Methods | | |
| Study design | 4 | Present key elements of study design early in the paper **[see introduction section]** |
| Setting | 5 | Describe the setting, locations, and relevant dates, including periods of recruitment, exposure, follow-up, and data collection **[Described in materials and methods; study site section]** |
| Participants | 6 | (*a*) Give the eligibility criteria, and the sources and methods of selection of participants **[see materials and methods; study design and population section]** |
| Variables | 7 | Clearly define all outcomes, exposures, predictors, potential confounders, and effect modifiers. Give diagnostic criteria, if applicable **[Described in materials and methods]** |
| Data sources/ measurement | 8* | For each variable of interest, give sources of data and details of methods of assessment (measurement). Describe comparability of assessment methods if there is more than one group **[described in materials and method section]** |
| Bias | 9 | Describe any efforts to address potential sources of bias **[Selection bias was avoided by employing a probability sample]** |
| Study size | 10 | Explain how the study size was arrived at **[see materials and method; sample size determination and sampling procedure]** |
| Quantitative variables | 11 | Explain how quantitative variables were handled in the analyses. If applicable, describe which groupings were chosen and why **[materials and method; data analysis section]** |
| Statistical methods | 12 | (*a*) Describe all statistical methods, including those used to control for confounding **[materials and method; data analysis section]** |
|  |  | (*b*) Describe any methods used to examine subgroups and interactions **[N/A]** |
|  |  | (*c*) Explain how missing data were addressed **[N/A]** |
|  |  | (*d*) If applicable, describe analytical methods taking account of sampling strategy **[N/A]** |
|  |  | (*e*) Describe any sensitivity analyses **[N/A]** |
| Results | | |
| Participants | 13* | (a) Report numbers of individuals at each stage of study—eg numbers potentially eligible, examined for eligibility, confirmed eligible, included in the study, completing follow-up, and analysed **[N/A]** |
|  |  | (b) Give reasons for non-participation at each stage **[N/A]** |
|  |  | (c) Consider use of a flow diagram **[N/A]** |
| Descriptive data | 14* | (a) Give characteristics of study participants (eg demographic, clinical, social) and information on exposures and potential confounders **[Results section; reported in Social demographic characteristics of participants sub-section]** |
|  |  | (b) Indicate number of participants with missing data for each variable of interest **[N/A]** |
| Outcome data | 15* | Report numbers of outcome events or summary measures **[Reported in data analysis section]** |
| Main results | 16 | (*a*) Give unadjusted estimates and, if applicable, confounder-adjusted estimates and their precision (e.g., 95% confidence interval). Make clear which confounders were adjusted for and why they were included **[Described in data analysis section]** |
|  |  | (*b*) Report category boundaries when continuous variables were categorized **[N/A]** |
|  |  | (*c*) If relevant, consider translating estimates of relative risk into absolute risk for a meaningful time period **[N/A]** |
| Other analyses | 17 | Report other analyses done—eg analyses of subgroups and interactions, and sensitivity analyses **[Results section; refer to table 4 and 5]** |
| Discussion | | |
| Key results | 18 | Summarise key results with reference to study objectives **[as described in discussion section]** |
| Limitations | 19 | Discuss limitations of the study, taking into account sources of potential bias or imprecision. Discuss both direction and magnitude of any potential bias **[as reported in discussion section]** |
| Interpretation | 20 | Give a cautious overall interpretation of results considering objectives, limitations, multiplicity of analyses, results from similar studies, and other relevant evidence **[as give in discussion section]** |
| Generalisability | 21 | Discuss the generalisability (external validity) of the study results **[This is reported in conclusion section]** |
| Other information | | |
| Funding | 22 | Give the source of funding and the role of the funders for the present study and, if applicable, for the original study on which the present article is based **[N/A]** |

*Give information separately for exposed and unexposed groups.

**Note:** An Explanation and Elaboration article discusses each checklist item and gives methodological background and published examples of transparent reporting. The STROBE checklist is best used in conjunction with this article (freely available on the Web sites of PLoS Medicine at http://www.plosmedicine.org/, Annals of Internal Medicine at http://www.annals.org/, and Epidemiology at http://www.epidem.com/). Information on the STROBE Initiative is available at www.strobe-statement.org.
